# Connecting healthcare professionals in Central America through management and leadership development: a social network analysis

**DOI:** 10.1186/s12992-020-00557-4

**Published:** 2020-04-15

**Authors:** Andrea M. Prado, Andy A. Pearson, Nathan S. Bertelsen, José A. Pagán

**Affiliations:** 1 INCAE Business School, P. O. Box: 960-4050, Alajuela, Costa Rica; 2 Department of Medicine, University of Minnesota Medical School, 401 East River Parkway, Minneapolis, MN 55455 USA; 3 Department of Public Health Policy and Management, School of Global Public Health, New York University, 715 Broadway, New York, NY 10012 USA

**Keywords:** Management, Leadership, Central America, Social network analysis

## Abstract

**Background:**

Leadership and management training has become increasingly important in the education of health care professionals. Previous research has shown the benefits that a network provides to its members, such as access to resources and information, but ideas for creating these networks vary. This study used social network analysis to explore the interactions among Central American Healthcare Initiative (CAHI) Fellowship alumni and learn more about information sharing, mentoring, and project development activities among alumni. The CAHI Fellowship provides leadership and management training for multidisciplinary healthcare professionals to reduce health inequities in the region. Access to a network was previously reported as one of the top benefits of the program.

**Results:**

Information shared from the work of 100 CAHI fellows from six countries, especially within the same country, was analyzed. Mentoring relationships clustered around professions and project types, and networks of joint projects clustered by country. Mentorship, which CAHI management promoted, and joint project networks, in which members voluntarily engaged, had similar inclusiveness ratios.

**Conclusion:**

Social networks are strategic tools for health care leadership development programs to increase their impact by promoting interactions among participants. These programs can amplify intergenerational and intercountry ties by organizing events, provide opportunities for alumni to meet, assign mentors, and support *collaborative action groups*. Collaborative networks have great value to potentiate health professionals’ leadership and management capabilities in a resource-constrained setting, such as the Global South.

## Background

The Consortium of Universities for Global Health (CUGH) recognizes the need to deliver global health education programs that also develop management capabilities [1]. Health-worker education traditionally occurs in medicine, public health, and nursing schools, but these organizations often do not provide the transformative leadership and management skills necessary for their students to address the complex challenges that health systems face [2]. Moreover, the transformational leadership model might need to be expanded to explain leadership in an international healthcare context [3]. The 21st^−^century education framework for health professionals by Frenk et al. [4] and the “No More Heroes Report” [5], among other scholars and organizations, have pointed to the need for strengthening management and leadership in the education of health professionals. In this regard, partnerships between business schools and health education institutions could be valuable given the former’s emphasis on practice and on fostering innovation [1].

Due to the magnitude of the challenges faced by health systems around the world, training in managerial and leadership skills would significantly improve health professionals’ abilities to become active agents of change. According to Harrison, Meyer, Chauhan, & Agaliotis [6] qualities around managing and making change, collaboration, continuous learning, balancing management theory and practice and leadership skills are required from health professionals to be effective, mainly when working in global contexts.

The concept of “social capital” denotes the social relations and resource advantages of individuals and communities [7, 8] and assumes that individuals use their network ties to pursue opportunities [9, 10]. It has been shown that individuals access a variety of resources held by other actors—particularly intangible ones—through interpersonal and inter-organizational relationships [11]. They acquire emotional support [12] and persistence [13]. Collaborative networks can be beneficial, among other things, to spread new knowledge and identify solutions to vexing problems that cannot be easily solved without teamwork and cooperation [11]. Networks also provide information and advice along the implementation process of a project [11]. Studies have shown that people consistently use their networks to get ideas, recognize opportunities, and tap into the strategic talent and market information (Freeman, 1999, [14]). Finally, those with whom individuals associate themselves also provide reputational and signaling content to resource holders (i.e., investors, donors) ([15, 16].

Leadership programs, seeking to develop leaders with a capacity to influence policy and bring about social and systems change, are increasingly interested in social networks to strengthen relationships among leaders in fields, communities, and organizations [17]. Thus, understanding the network structure and interactions would allow academics, directors, and donors to make decisions on how to design these programs and which activities to support. By becoming a member of the right health professional network, individuals can have access to different types of resources—including emotional support [12].

The Central American region is a diverse and complex region that faces many health care access and population health challenges. The latest “Health in the Americas+” report [18] describes recent achievements of the countries in the region, such as substantial increase in life expectancy at birth in Guatemala and marked reduction in infant mortality in El Salvador. The report also describes many of the challenges unique to each country, including the prevalence of chronic health conditions and rising costs of medical care in Costa Rica, language access barriers in Guatemala, needs to strengthen primary care in Honduras, and rural-urban health disparities in Panama. Solutions for the health care and public health problems facing the region require not only financial resources, but also the development of professionals with the necessary leadership and managerial skills to navigate multiple sectors, innovate, and design and execute effective health intervention programs.

The Central American Healthcare Initiative (CAHI) Fellowship is a leadership development program that seeks to create the next generation of Central American leaders who can improve health systems’ performance and population health. Details about CAHI’s structure have been previously reported (see [19] for a thorough description of the program, its design, rationale and accomplishments during the first 3 years). Education and training programs seeking to develop leaders with a capacity to influence policy and bring about social and systems change are increasingly interested in social networks to strengthen relationships among leaders in different fields, communities, and organizations [17]. Leadership networks have the potential to provide members with resources and support, while increasing the scope and scale of their individual and collective impact [17]. Central American countries have often worked together as a region, whose geographic and historical backgrounds inherently support the development of such networks. Understanding the network structure and interactions among the CAHI Fellowship alumni would allow us to assess the real impact of collaboration and teamwork within CAHI’s alumni.

The CAHI Fellowship illustrates a successful example of a social network in its leadership and management training to health professionals, which actively promotes collaboration among alumni from multiple sectors and countries. This study explored the interactions among the CAHI Fellowship alumni network, and associated impact factors for this network to improve population health within respective fellows’ communities. Its results can be useful to inform other leadership programs such as the “Improving Global Health fellowship” at the National Health Service [20] or the “Healthcare Management Programme” at Strathmore Business School in Kenya [21].

## Method

### The Central American Healthcare Initiative (CAHI) fellowship

Investment in leadership programs for physicians and other health professionals in low- and middle-income countries is limited. However, some organizations have developed training and networking programs to strengthen these capabilities among health professionals. Some examples include: a) the Young Physician Leaders (YPL) launched by InterAcademy Partnership for Health for health professionals worldwide [22], b) the international network for Human Resources for Health (HRH) managers in nine Francophone African countries developed by Vision Tokyo 2010 [23] and c) the Flagship Program, a partnership between the World Bank and the Harvard TH Chan School of Public Health [24]. These programs focused on capacity building, knowledge transfer, networking and peer learning, among other things, but did not carry a specific Central American focus.

The CAHI Fellowship is an executive education diploma program for multidisciplinary health professionals in Central America. The program seeks to equip participants with the technical skills needed to manage innovative projects successfully as well as provide fellows with the necessary practice experiences in leading teams and change processes so that they become active social change agents in their communities. In 2012, the faculty of the Center for Latin American Competitiveness and Sustainable Development (CLACDS, by its Spanish acronym) at INCAE Business School began working with the staff and executive board of CAHI to design a fellowship program for Central American health leaders from the public, private, and non-profit sectors with a broad range of professional expertise. CAHI, a non-profit organization based in the United States, was formed to support health professionals in Central America, as they strengthen their skills in leadership, innovation, and project management.

CAHI and CLACDS launched the CAHI Fellows program in 2013. The program provides health professionals with different levels of education and experience the opportunity to improve their leadership and innovation skills while they apply project management theory directly to a specific project in solving a health-related challenge in the region. The fellowship consists of four one-week in-person modules over nine months, where participants receive 175 h of coursework. Additionally, participants engage in virtual sessions to refine the work plan of their projects, receive one-on-one coaching from faculty, and learn about emerging issues in health innovation. Coursework includes team-building activities, simulations, workshops, case studies, and theory-focused lectures.

Five cohorts of CAHI Fellows graduated between 2013 and 2019. They all became part of the CAHI Fellows’ Network—a multidisciplinary network of professionals who share the common goal of reducing health inequities in Central America. Tables 1 and 2 provide descriptive statistics of CAHI’s network, including information on the different projects carried out by fellows organized in four categories. By February 2019, the network had 100 fellows distributed in Central America, with representatives from the government, private sector and civil society. CAHI promotes knowledge exchange among alumni from different generations as well as countries and helps strengthen their ties.

**Table 1:** Descriptive statistics of fellows

	2014	2015	2016	2017	2018	Total
Total	16	21	20	24	19	100
Country						
Guatemala	1	3	5	6	5	20
Honduras	1	2	3	3	3	12
El Salvador	2	3	2	3	1	11
Nicaragua	5	3	2	4	2	16
Costa Rica	6	7	7	5	4	29
Panama	1	3	1	1	4	10
Mexico	0	0	0	2	0	2
Gender						
Male	11	11	10	11	8	51
Female	5	10	10	13	11	49
Age group						
21-30 years	6	1	3	4	7	21
31-40 years	4	10	6	11	3	34
41-50 years	6	5	7	5	7	30
51-60 years	0	3	4	2	1	10
More than 60 years	0	2	0	0	0	2
Non specified	0	0	0	2	1	3
Health Professional background						
No	4	10	6	6	6	32
Yes	12	11	14	18	13	68

**Table 2:** CAHI Fellows’ project type

Project type	2014	2015	2016	2017	2018	Total	Example
Hospital management executive training	4	8	9	12	4	37	Text messages for the prevention of hypertension Intersectional plan for the prevention of violence, unwanted pregnancies, and chronic diseases in the adolescent population
Strategies for health promotion and prevention	3	6	6	5	4	24	Delivery of high-quality, low-cost generic drugs in rural areas Mobile platform technology to provide remote consultations and medical care
Access to medical services	5	4	3	3	8	23	Plan to improve processes to reduce surgery waiting list in a public hospital Information systems medical imaging to improve response time to diagnose patients
Training for health professionals	4	3	2	4	3	16	Specialization program focused on patient safety for nurses Guidelines for training health professionals in the care of indigenous populations

CAHI Fellows have developed relationships among themselves, both on their own initiative, as well as intentionally promoted by CAHI. The Central American region provides certain conditions that can support the development of networks. For instance, given that the region encompasses six countries in a small territory, public and private organizations have often felt the need to reach out to others to increase the volume of its operation. Sharing the same main language (Spanish), similar historical background (indigenous cultures and colonial period) and a precedent of efforts seeking geographic, economic and political integration (Central American Integration System), also support these regional efforts. In the health sector, countries have articulated initiatives such as the Central American Council of Health Ministers that coordinate efforts at the regional level (e.g., jointly bargaining and purchasing health supplies to access a more favorable price due to higher volume). Likewise, multilateral and cooperation agencies (e.g. Pan-American Health Organization, Spanish Cooperation Agency), have often had a regional approach to support interventions in multiple countries. The development of regional working groups—particularly when there was a high flow of aid into the region—precedes the CAHI fellowship. Unfortunately, once the integration efforts vanished and international aid left, so did many of these regional initiatives. Once more, a non-profit organization like CAHI brings a regional vision into its intervention: the fellowship program.

The ties developed among CAHI fellows go beyond belonging to the same cohort and being classmates. Sharing information outside of the class, supporting other CAHI fellows through mentorships, and developing projects together are some of the most common ways the fellows interact. As such, we mapped these networks to understand better how CAHI management can support and strengthen the development of its alumni network. CAHI believes that fellows connected through strong ties will be more effective than individual fellows when it comes to making significant improvements in the health of the region, expand health care access and delivery.

Despite the similarities that the CAHI fellowship shares with other programs for human resources in health, some features make it different. First, its interdisciplinarity as it selects representatives from different sectors and backgrounds (e.g., public, private and civil society), as well as from different professional backgrounds (e.g., information systems and industrial engineers, lawyers, pharmacists, business administrators). Second, CAHI fellows are recruited with a project proposal that they have to advance during the program. Thus, they can apply/practice in their own project the acquired capabilities. Finally, the program is done in partnership with a business school. Business schools can contribute to the training of health leaders on innovation management and entrepreneurship, among other skills that would help them focus on implementation rather than on research [25].

We used social network analysis [26, 27] to conduct our study. Dershem, Dagargulia, Saganelidze, & Roels [28] propose a set of steps to develop a network analysis. First, a study design phase that includes determining and compiling a complete list of the population, defining the critical types of relationships among them, and a timeframe for the analysis. Second, a data collection phase in which the research team develops the questionnaire and initiates the data collection process. Then, the data collected must be structured in a way that can be analyzed using social network analysis and data visualization software.

We identified the relationships between the total population of CAHI Fellows (*n* = 100). For this population, we mapped three types of relationships: information-sharing between fellows, mentorship, and the development of joint projects among fellows. The difference between mentorship and joint projects is that the former is assigned by CAHI management, while the latter is an initiative that two or more fellows start voluntarily because they see a potential benefit from collaborating. Table 3 shows the definition of the different types of ties and examples within each category.

**Table 3:** Definition and examples of network ties

	Definition	Example 1	Example 2
Information sharing	Exchange information by email, telephone, text messages, face-to-face get-togethers, public events, online meetings, webinars, or field visits. *Note:* CAHI organizes multiple events to support these sharing opportunities.	Director (MD) of one of the principal public hospitals in Panama (Gen_5) visited the Project Coordinator (nurse) of an Ambulatory Care Hospital in a public hospital in Costa Rica (Gen_4). The intention was to learn from her experience to replicate the project in Panama, including information about the implementation challenges; financial, infrastructure and human resources requirements; and lessons learned during launching and first months of the hospital.	An industrial engineer from Honduras (Gen_3), an MD from El Salvador (Gen_1), were the speakers of a panel discussion (online) where they both presented their projects on health interventions for teenagers to the CAHI fellows. As part of the preparation, they shared information about their projects, including strategies on how to approach teenagers at social risk, and the challenges of partnering with organization from different sectors (government, NGO, and for-profit organizations)
Mentorship	An assignment to support a CAHI fellow by providing guidance and recommendations based on personal knowledge and experience. *Note:* CAHI invites particular alumni to become mentors based their expertise on a specific topic, potential value-added for a fellow’s project, and mentees’ expressed needs	Costa Rican information systems engineer (Gen_1) mentored technology focus projects in during multiple cohorts.	Costa Rican industrial engineer working in public hospitals (Gen_2) mentored a Panamanian MD working at the social security (Gen_5) leading an initiative to decrease waiting lists in public hospitals
Joint projects	Coordination of efforts and resources for the implementation of joint projects or initiatives.	A Costa Rican psychologist working as Executive Director of a nonprofit organization promoting healthy behaviors in teenagers via an online platform (Gen_2) and a Costa Rican MD working as a Social Security Director of a health region (Gen_1) are partnering to promote the use of the platform to prevent risky behavior in this geographic area—where violence and poverty are higher than in the rest of the country.	Costa Rican information systems engineer working as the Social Security Medical Record Director (Gen_4) and the Program Coordinator of a public palliative care hospital implemented a project for remote patient care using high technology devices and telemedicine systems.

We also identified three characteristics of the network members: country of origin, professional background, and type of project developed. We modeled the network as a one-mode directed social network, meaning all nodes are individuals with the same characteristic (i.e., they are all CAHI fellows). We included only direct ties between the nodes. For instance, when one fellow exchanged information with another member directly, we captured it on the map. We did not include cases when one fellow exchanges information with all classmates via email.

We collected data about the direct relationships among CAHI fellows in several ways. First, the CAHI management team met and discussed all the relationships that they knew have happened among fellows. Given that the CAHI management team spent a significant amount of time with the fellows during the program and followed up on their project development during this time, it was not difficult to keep track of these connections, as the cohorts were relatively small. Moreover, many fellows would call and report back to CAHI management on their project progress years after they graduated, which was also captured in our program evaluation. Second, we revised archival data on mentorship assignments through different cohorts. Third, we distributed a survey among the fellows where they had to validate the relationships identified by the CAHI management team and asked them to report on any other relationship with fellows from all generations. The survey was distributed online. A draw of a free trip to attend a CAHI event in Panama was offered among the alumni who responded to the survey within a given amount of time. The response rate was 58%.

We coded the data on three kinds of relationships among fellows—information sharing between fellows, mentorship, and joint projects among fellows.

We took two complementary approaches for analyzing these network ties as identified: visual and statistical. The visual approach provided a comprehensive and explorative perspective on the network. The statistical approach provided intuitive metrics that captured the salient features of the network [29]. These two methods helped us understand the characteristics of the network and actors (e.g., density, inclusiveness, average degree) [28].

The visual analysis was conducted using the *Gephi* software [30]. For each map of each type of relationship and the different characteristics of the network members, we used *Force Atlas* [31] to compact the figure, considering that nodes become closer if they are connected [29]. The size of the node (i.e., size of the circles) captures the number of connections per individual. For the statistical analysis, we used a software called *Ucinet 6* [32], to estimate several measures (density, inclusiveness, average degree) to better understand the characteristics of the network and each type of relationship between the individuals [28, 29].

## Results

**Fig. 1:**
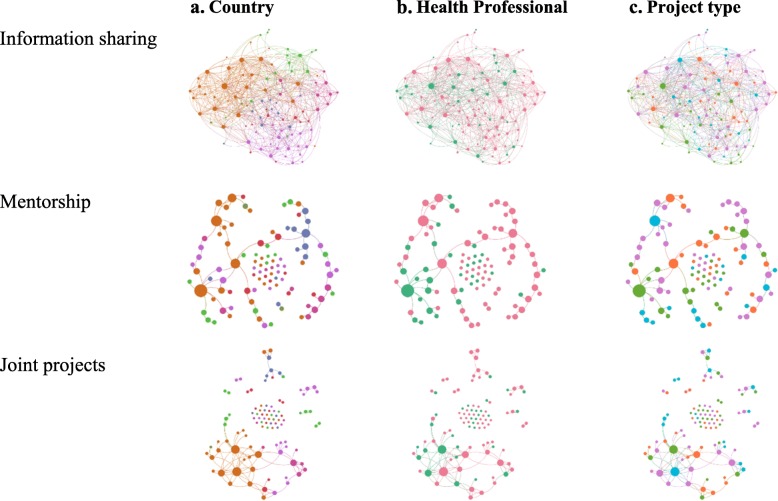
CAHI network maps. **a**. Country: Costa Rica (Orange), Guatemala (Purple), Nicaragua (Green), Honduras (Red), El Salvador (blue), Panama (Dark pink), Mexico (Dark Green). **b**. Health Professional: Yes (red), No (green). **c**. Project type: Reinforcement of hospital management (purple), Strategies for disease prevention and health promotion (Green), Access to medical services and prescription (orange), Training for health professionals (blue)

Figure 1 presents the network maps describing how CAHI fellows shared information among themselves, engaged with mentors, and developed joint projects. The results from the maps are presented according to three fellow characteristics: country of origin, professional background, and project type.

### Information-sharing network

These maps show how all CAHI alumni are engaged in sharing information. Figure 1a shows how these actions are more frequent among fellows from the same countries. Figure 1b and c indicate that CAHI fellows shared information with individuals from different professional backgrounds and project types. Having representation from all Central American countries among CAHI’s cohorts has always been intentional in the selection process. For three generations (from second to fourth cohorts), CAHI was also intentional on having a group of fellows in the cohort working in hospital management projects. However, in the last two generations, the selection process gave preference to fellows whose projects focused on primary care.

### Mentorship network

Mentorship assignments were intentionally made by CAHI management. The process for selecting mentors and matching fellows to mentors evolved with time. In the early years, mentors were professionals from different countries with relevant expertise in the area of interest of each fellow (results not reported here). It was not until the third year, and once that there was a critical mass of expertise among the alumni that CAHI decided to assign alumni as mentors to the current fellows.

Mentors were assigned according to the area of expertise most suitable to a given project from a fellow. Thus, one observes more clustering around professions (1b) and project type (1c) than around the country (1a). The mentorship network map also identifies a group of CAHI fellows that did not connect in this form with other fellows (see unlinked points in the middle).

### Joint projects network

Joint projects are a step further than information-sharing and mentorship type of relationship. Joint projects often demand to have a shared agenda, resource assignment (e.g., time allocation, financial resources, formal partnerships), or high coordination efforts at the organizational level. Differently than in the case of information-sharing and mentorship relationships, CAHI does not intentionally support the development of specific joint projects, nor provide financial support to the participating fellows. Thus, the fellows engaged voluntarily in these efforts.

Figure 1a shows the clustering of the network by country. Coordinating efforts with fellows from the same country is likely to be more accessible not only logistically, but also in terms of identifying similar needs to address in a joint project. Figure 1a and c show that professionals from different backgrounds and project type coordinated these joint efforts. Such observation suggests that CAHI fellows are engaged in projects with multidisciplinary teams and using different intervention approaches (i.e., project types) to address the challenges in health and health care.

CAHI does not directly provide financial support for these projects. Most of them depend on the resources of their institutions (e.g., financial, time allocation, contact networks), as these joint projects become a mechanism to achieve the goals of each organization. When they are for-profit entrepreneurial initiatives, they bootstrap. Grants from local governments are scarce. The international community focused mainly in the Northern Triangle (i.e., El Salvador, Guatemala and Honduras) might be supportive of cross-border or joint projects. So far though, most joint projects are among fellows from the same country and they have yet received funds this way. Thus, CAHI fellows are likely to develop joint projects after participating in the program, as it allows them to meet potential partners and discover collaborating opportunities.

Table 4 presents the statistical results from the network analysis. The information-sharing network has a score of 100 for inclusiveness (i.e., all CAHI fellows are engaged in this activity one way or the other). This level of inclusiveness is higher than in the mentorship and shared project networks. These last two networks have similar inclusiveness ratios, despite CAHI management intentionally promoting the mentorship relationships, while joining joint projects was voluntary. The joint project network exhibits smaller density and average degree indicators than the information-sharing network, but higher than the mentorship network, reinforcing the idea that many of the fellows have the self-motivation to engage with other fellows and develop joint projects.

**Table 4:** Network descriptive statistics

	Number of members	Number of ties	Density^a^	Inclusiveness^b^	Average Degree^c^
Information sharing	100	588	5.9	100	5.88
Mentorship	100	75	0.8	75	0.75
Joint projects	100	114	1.2	76	1.14

^a^The number of actually-occurring relations or ties as a proportion of the number of theoretically-possible relations or ties

^b^The percentage of nodes that are connected to other nodes in the network. The more nodes are isolated (no connection to any other nodes in a network) the lower the inclusiveness

^c^Sum of the total of ties that each actor has by the total of actors in the network

## Discussion

Central American countries face critical health care and population health challenges. Potential solutions to these challenges require financial investments, as well as the need to develop effective collaborative networks within and across countries in the region. These collaborative networks can facilitate knowledge dissemination and promote teamwork and cooperation.

We analyzed three networks from a leadership development program—the CAHI Fellowship—to understand the relationships among the five alumni cohorts. CAHI fellows strongly believe that “having access to a network of innovative leaders” is the main benefit derived from this program [19], and this perception matches what entrepreneurs globally believe to be the most critical benefit they obtain from participating in learning programs (e.g., accelerating the development of new ideas and projects) [33].

The role played by CAHI management in strengthening the networks of fellows is also salient. For instance, the selection process is key to achieving robust information-sharing and an active joint project network because it deliberately promotes diversity in terms of country of origin, professional background, and types of projects conducted during and after the completion of CAHI Fellowship activities. Organizing events and providing opportunities for alumni to meet and get to know each other (e.g., panel presentations, in-country meetings and webinars) are central activities to the development of intergenerational and intercountry ties. These events can also help strengthen the ties among the same country alumni, which may lead to the development of new projects in the future.

A diverse cohort in terms of project type, for example, provides the opportunity for physicians working in hospitals to exchange information with other professionals working at the local level (e.g., community health workers). These interactions help to increase the level of understanding of the challenges faced by many fellows in different contexts. The CAHI Fellowship seems to provide an optimal space for a group of diverse individuals to interact, allowing them to transcend hierarchies or status barriers within and across professions.

A mentorship component can become part of a network development strategy within leadership programs. The development of these relationships can rely not only on the management team assigning alumni with relevant expertise to the new fellows but also on new fellows directly requesting an introduction to alumni they think might be a good fit for them. Something we learned along the years was that even when the mentorship is assigned in terms of expertise fit, the more personal connections (e.g., ability to work together and time availability) between fellows cannot be guaranteed by CAHI management. CAHI is not compensating the mentors for their time, so it is difficult to demand dedication beyond what they voluntarily want to provide. Thus, it might be worth for new fellows to have more than one alumni mentor, not only to increase the inclusiveness of the network, but also to have a backup in case a personal connection does not work.

Finally, network maps show fellows engaging in the development of joint projects. If CAHI selects fellows exhibiting high motivation, commitment, and entrepreneurial drive—among other features—it increases the probability of them pursuing joint projects on their own after program completion. Therefore, the selection process becomes a strategic step for CAHI—or any other leadership program—to extend its impact beyond the training component and into the generation of joint projects.

Skill-acquisition and implementation of individual projects could be an entry point instead of an end by itself in leadership development programs like the CAHI Fellowship. Fostering strong network ties among alumni can lead them to join efforts in collectively achieving social impact through policy changes. Leadership development programs could benefit from assigning resources to invest in the development of these networks and not solely in the training component of the program. Besides, these programs could use frameworks such as the Toronto Health Organization Performance Evaluation (T-HOPE) [34] as a monitoring and evaluation mechanism, that includes measures that balance feasibility, comparability and credibility.

Investing in the platforms and spaces where alumni can meet and interact with each other is also essential to strengthen the network. In this regard, these programs should not underestimate the power of face-to-face interactions, vis-à-vis virtual connections. Moreover, leadership programs can support the formation and operation of *collaborative action groups,* where fellows from the same country or with the same project type can connect. To support these groups, the program’s management team can organize events periodically for fellows to have working meetings and provide administrative support to facilitate their participation in joint projects such as scheduling and needs assessment.

Leadership programs could provide financial support to joint projects developed by network members. Alternatively, they can seek to establish connections between the impact-investing communities and these projects. These investors place financial resources in projects or enterprises that generate social or environmental goods, services, or benefits, combining philanthropic objectives with mainstream financial return Höchstädter and Scheck [35]. It might not be challenging to find impact-investors interested either in a specific region (e.g., Latin America) or a specific area (e.g., health care). The main challenge would be to bring joint projects to a development stage, where they become attractive to investors and where the fellows can evaluate their impact. Systematic efforts towards impact measurement is also relevant for CAHI as an organization, due to the importance donors are giving to this type of assessments.

There are important limitations in this study. The analysis constitutes a “photograph” of the network by mid-2019, missing a temporal dimension to control for the time of entry of the fellows. The paper does not explore the quality of the ties that were formed during and after participation in the CAHI Fellowship (e.g., what kind of information members exchanged, what type of projects they are developing together). Also, the study did not go into detail on the profiles of those individuals that are most central in the network. Analyzing these profiles could provide valuable insights to inform the selection process, providing an interesting avenue for future research. Finally, a major limitation for CAHI was the difficulty to promote sustainable fundraising to maintain the program. While it was very important to create CAHI as a valuable educational opportunity made free for its fellows, the goal to utilize the CAHI network to identify diversified and sustainable funding resources to ensure longevity of the program was found to be much more challenging.

## Conclusion

Social networks are essential tools to expand knowledge and learning within leadership development programs. Similarly, network-building activities can be considered an essential deliverable of these programs and in turn, help strengthen collective efforts to improve health equity across all populations in Central America. Indeed, the creation of such a network of leaders was found to be a major outcome itself. Our network analysis of the CAHI leadership development program showed that the main benefit of the program is having access to a network of innovative leaders that can help network members advance learning and the development of new ideas. Interactions among a diverse group of professionals help participants to understand and address different healthcare challenges throughout the region more quickly and effectively.

## Data Availability

The datasets during and/or analyzed during the current study are available from the corresponding author on reasonable request.
